# Single left coronary artery with separate origins of proximal and distal right coronary arteries from left anterior descending and circumflex arteries – a previously undescribed coronary circulation

**DOI:** 10.1186/1749-8090-2-20

**Published:** 2007-04-20

**Authors:** Pankaj Kaul, Kalyana Javangula

**Affiliations:** 1 Department of cardiac surgery, Yorkshire Heart Centre, Leeds General Infirmary, Great George Street, Leeds, LS1 3EX, UK

## Abstract

A single left coronary artery with right coronary artery arising from either left main stem (LMS) or left anterior descending artery (LAD) or circumflex artery (Cx) is an extremely rare coronary anomaly. This is the first report of separate origins of proximal and distal RCA from LAD and circumflex arteries respectively in a patient with a single left coronary artery. This 57 year old patient presented with unstable angina and severe stenotic disease of LAD and Cx arteries and underwent urgent successful quadruple coronary artery bypass grafting. The anomalies of right coronary artery in terms of their origin, number and distribution are reviewed.

## Case presentation

**Figure 1:**
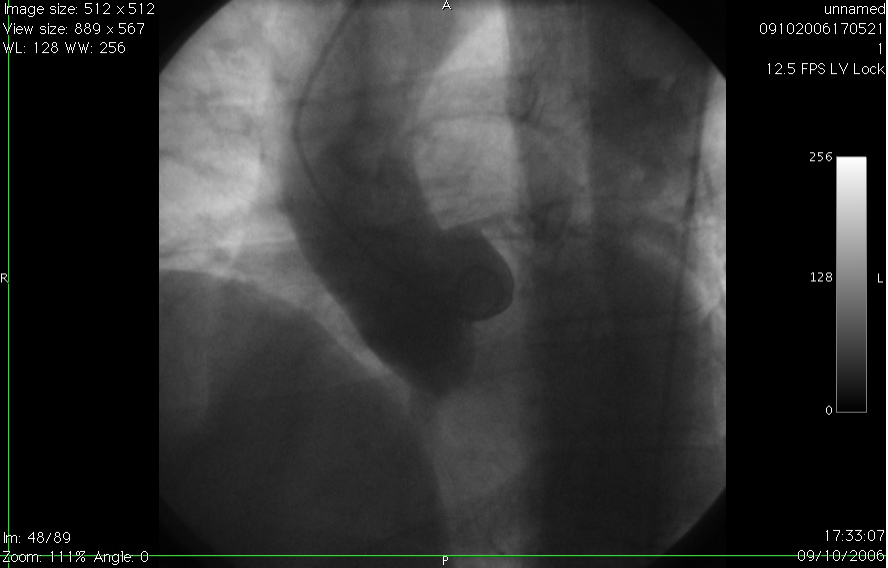
Aortic root angiogram showing single left coronary artery and absent rightcoronary ostium.

**Figure 2:**
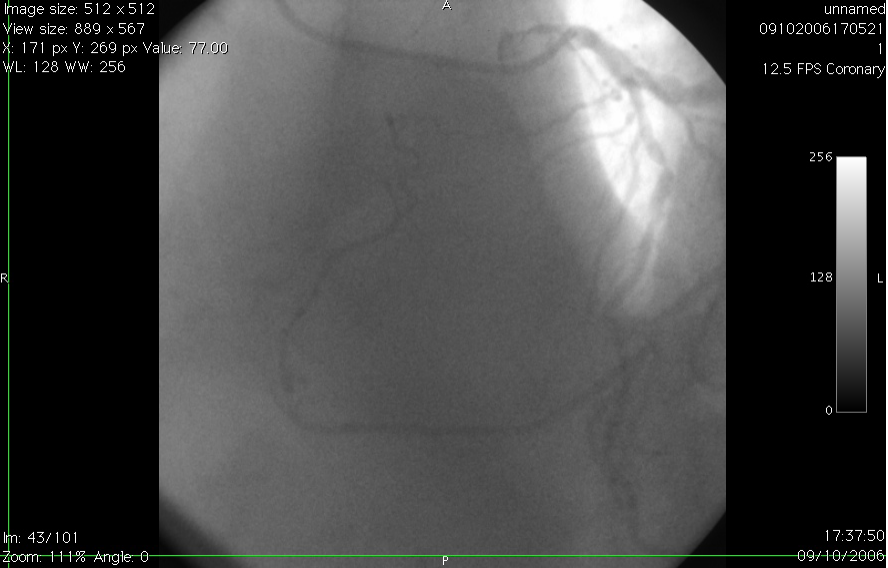
Left coronary angiogram in LAO view showing anomalous origin ofproximal RCA from proximal LAD, proximal to LAD stenosis.

**Figure 3:**
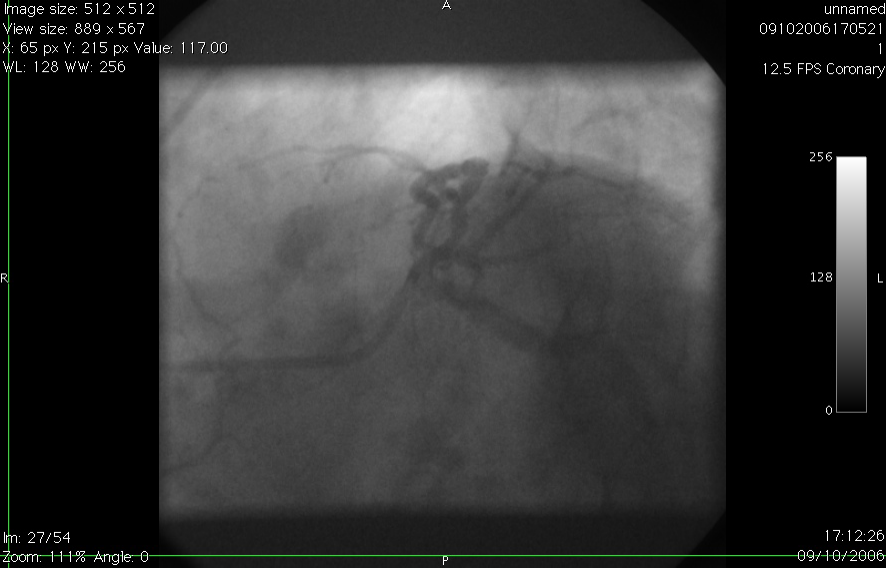
Left coronary angiogram in cranial view showing the anomalous origin of proximal RCA from proximal LAD.

**Figure 4:**
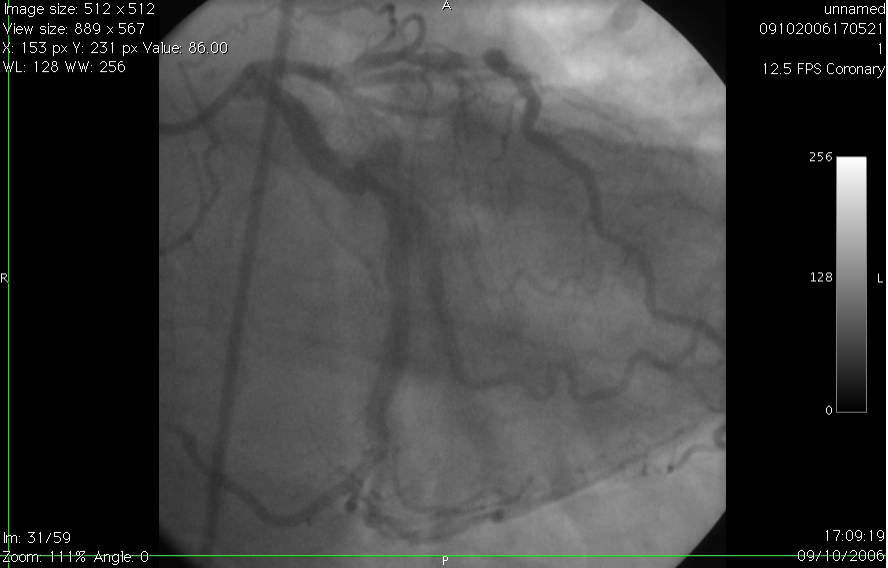
Left coronary angiogram in RAO view demonstrating the proximally stenosed circumflex artery continuing as the distal right coronary artery after giving off the large obtuse marginal and posterior descending arteries.

**Figure 5:**
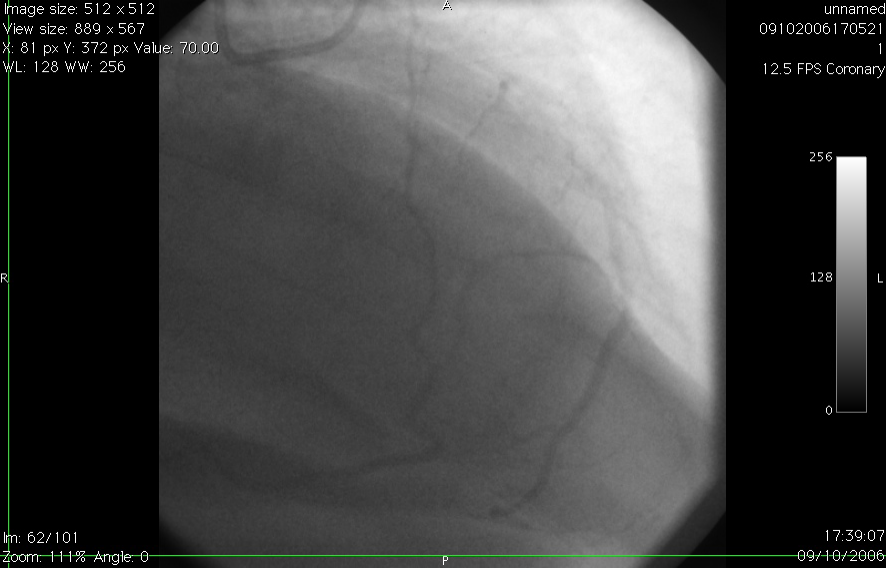
The smaller anomalous proximal right coronary artery arising from LAD establishes an end to side anastomosis with the larger distal RCA arising as a cotinuation of the circumflex artery. The distal RCA beyond the anastomosis seems to meander along proximally.

A 57 year old male presented with unstable angina. Risk factors included hypertension and hypercholesterolaemia. Significant past history included multiple episodes of deep vein thrombosis and pulmonary embolism. Coronary angiography demonstrated a single coronary artery arising from left coronary sinus (fig 1) which divided into a normal sized left anterior descending artery (LAD) and a circumflex (Cx) artery. LAD had a 99% stenotic lesion beyond the first septal and was a good sized vessel going just beyond the left ventricular apex (Fig 2). From its proximal segment, beyond the origin of first diagonal and prior to the origin of first septal artery, arose 3 right ventricular branches, the largest of which crossed the right ventricular outflow tract (RVOT), 2 centimetres below the pulmonary valve, to gain the anterior right atrioventricular groove (Fig 2, 3), descended in the groove to anastomose with the distal right coronary artery (Fig 2), which arose as a continuity of the circumflex artery (Fig 4), as described below. The second right ventricular branch crossed the RVOT below the first but petered out well before it could gain the anterior right AV groove. The third branch supplied the right ventricle and followed a course close to the LAD (fig 2). The diagonal artery was a large bifurcating artery with significant proximal stenosis. The circumflex artery had severe stenosis proximally after which it gave a large obtuse marginal branch and then the PDA in the posterior interventricular groove and thereafter continued in the AV groove as the right coronary artery (Fig 4). This right coronary artery then gave off a ventricular branch to the inferior surface of the right ventricle and thereafter anastomosed with the proximal RCA arising anomalously from the LAD as described above (Fig 5). There was no stenotic lesion in this composite, anomalously arising proximal and distal right coronary arterial system.

**Figure 6:**
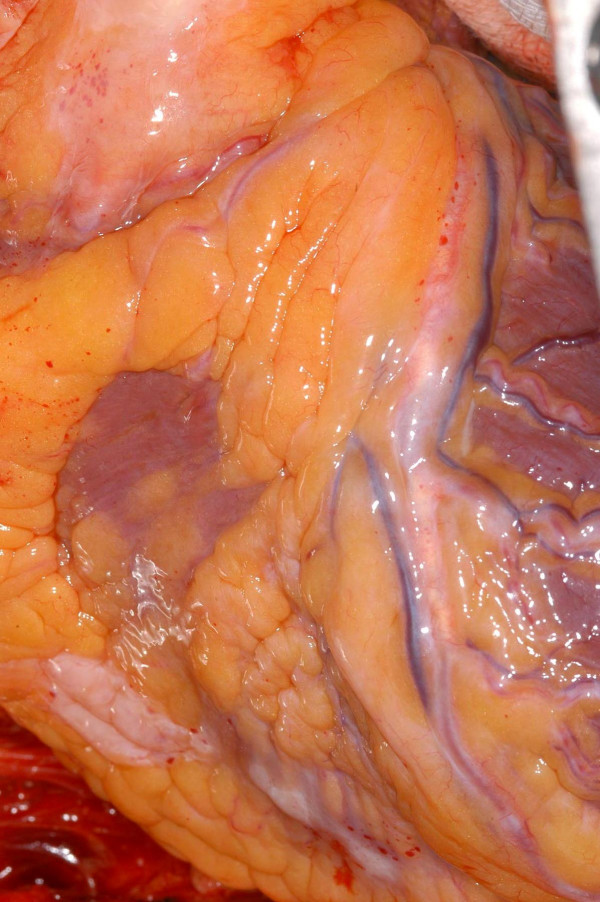
The middle of the three right ventricular branches from LAD supplying the right ventricle. The anomalous proximal right coronary artery arose from LAD above this branch and is not seen.

**Figure 7:**
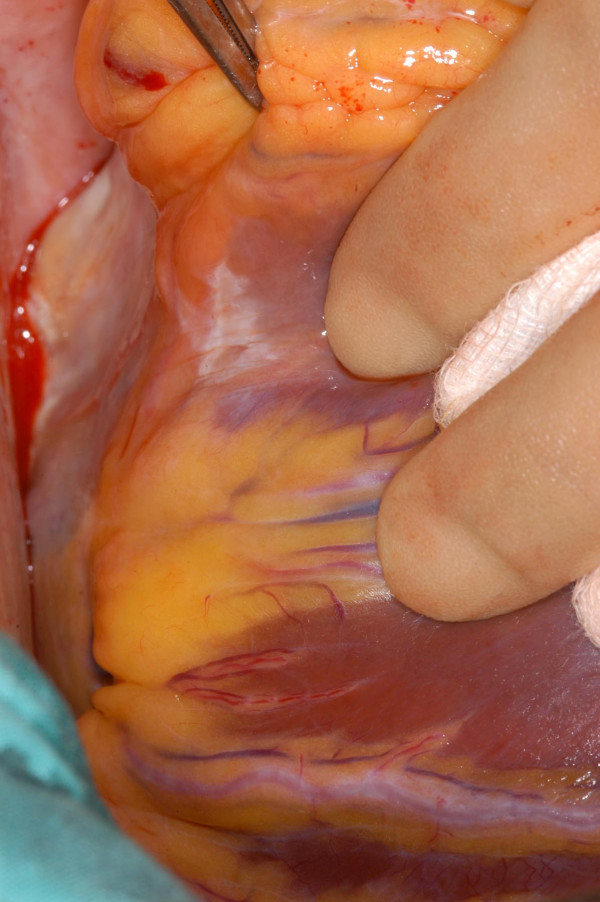
The posterior descending artery is seen to arise from the circumflex. A smaller branch from the anomalous distal RCA, seen above the PDA in the picture, supplies the inferior right ventricular surface.

Patient was taken to theatre for urgent CABG. Operative findings confirmed the following: Proximal RCA was arising as a branch from the proximal LAD after the first diagonal, crossed the RVOT and gained the anterior aspect of the right AV groove to anastomose with the distal RCA which arose as a continuity of circumflex artery. LAD gave off two further branches to supply the right ventricle (Fig 6). The distal circumflex continued to the crux of the heart, gave off PDA in the posterior interventricular groove (Fig 7) and thereafter continued as RCA till just above the acute margin of right ventricle. This distal RCA also gave off a right ventricular branch to the inferior right ventricular surface below the acute margin of the heart (Fig 7). Employing cardiopulmonary bypass, with antegrade cold blood cardioplegic arrest, quadruple coronary artery bypass grafting was performed. Left internal mammary artery (LIMA) graft was anastomosed to LAD and separate saphenous vein bypass grafts were constructed to Dx, OM Cx and PDA from Cx. Bypass was discontinued easily in sinus rhythm, without ionotropes and patient transferred to ICU in a satisfactory haemodynamic condition. He was transferred to ward on first postoperative day and home 8 days after surgery.

## Discussion

In a study of 126,595 patients who underwent coronary angiography over a period of 28 years, from 1960 to 1988, at Cleveland Clinic, Yamanaka et al reported coronary artery anomalies in 1686 (1.3%) patients. 1461 (87%) had anomalies of origin and distribution, and 225 (13%) had coronary artery fistulae [1]. After detailed coding of coronary angiograms in 24,959 patients, CASS study identified major coronary anomalies in 73 (0.3%) patients, out of which only 15 (0.06%) involved right coronary artery, with 7 out of 15 such coronary arteries coursing between the great vessels [2]. In a retrospective analysis of the angiographic data of 5253 consecutive adult patients in a Turkish population, only 5 (0.09%) had anomalous origin of right coronary artery, either from left coronary ostium (0.03%) or from above left coronary ostium (0.06%) [3]. Among 4100 adult patients in a North Indian study, 39 (0.95%) patients had one or more anomalous coronary arteries, with right coronary artery the commonest anomalous vessel, being involved in 19 (0.48%) patients, and arising from left sinus of Valsalva in 15 and non-facing aortic sinus in 4 [4]. In a large study of 13010 adult patients in Florida where 41% patients were of Hispanic origin, 80 (0.61%) patients had anomalous coronary circulation, out of which 50 (0.37%) had anomalous origin of right coronary artery, with 35 arising from left aortic sinus, 14 from posterior sinus and 1 from left coronary artery [5]. In a retrospective analysis of 4094 Chinese patients from Shanghai who had undergone diagnostic coronary angiography, 32 (0.78%) had anomalies of coronary origin, the right coronary artery being involved in 21 (0.5%) patients, originating from left sinus in 15 and non-coronary sinus in 2 [6]. Interestingly, both this and the previous study showed that the anomalous coronary arteries were not at a higher risk for the development of coronary atherosclerosis. Also, all the above studies from across the globe, show a relatively constant incidence of coronary anomalies at less than 1.5%, with right coronary anomaly being the commonest except in one study [2] where circumflex artery was the commonest anomalous vessel.

Right coronary anomalies can involve origin or distribution of the artery. When right coronary artery arises anomalously from the left coronary sinus [1-6], it often pursues a course between the two great vessels and is especially prone to compression during ventricular diastole. This malignant right coronary anomaly is uniquely demonstrable by multi-slice CT coronary angiography [7] or cine MR imaging [8]. Benge et al demonstrated significant systolic compression of such an anomalous RCA arising from left sinus with significant cardiovascular morbidity [9]. Right coronary artery does rarely arise from the non-coronary sinus of Valsalva [1-5] and from above the non-coronary sinus [10,11] and although, in themselves, both these anomalies do not predispose to malignant compression, they do have implications on percutaneous interventions should the anomalous coronary artery become involved with atherosclerosis. There has been at least one report of the origin of right coronary artery from below the aortic valve [12]. An abnormally high take off of the right coronary artery from above the sinotubular line with an anomalous intraaortic initial course with implications for angiography, angioplasty and surgery has been described [13-15]. Right coronary artery origin from descending thoracic aorta has been described at autopsy in an infant with hypoplastic left heart syndrome [16]. Right coronary artery can arise from main pulmonary artery in association with other congenital anomalies. Most patients with isolated origin of right coronary artery from main pulmonary artery remain asymptomatic but may develop myocardial ischemia and sudden death, and, therefore, reconstitution of double ostium coronary system is recommended [17,18]. Lessick et al reported the diagnosis of the anomalous origin of a posterior descending artery from the right pulmonary artery by multidetector CT angiography [19]. Grunenfelder reported right coronary artery arising from pulmonary trunk in a patient with AP window presenting without myocardial ischemia, due to the presence of left to right shunt [20].

Double right coronary arteries have been anecdotally reported. They can be different to differentiate from a high take off of a large right ventricular branch [21], both the arteries can be involved with atherosclerosis [22] and the two coronary arteries can give anatomically diverse branches [23].

A single left coronary artery arising from the left coronary sinus is a rare anomaly with right coronary artery arising from either the left main stem or the branches downstream from the main stem. If arising from left main stem, as also when arising from a separate ostium in the left coronary sinus, the anomalously arising right coronary artery can course anterior, posterior or in between the great arteries, the last course constituting a malignant type of anomaly due to extrinsic compression. Arteaga described the third case of the Shirani-Roberts subtype IB4 anomaly with the right coronary artery arising from the left main stem and coursing behind the aorta to reach the right heart [24]. Ajith et al described a post-infarction VSD in a patient with single left coronary artery with right coronary arising from left main stem who had significant lesions in left anterior descending and right coronary arteries [25]. Lopushinsky surgically reimplanted the right coronary artery into aorta in a man who had right coronary artery arising from left main and coursing thereafter between the great arteries associated with external compression and ischaemic symptoms [26].

Right coronary artery arising from left anterior descending artery in the absence of a normally situated right coronary ostium is considered a variant of single left coronary artery and ten such cases have been described in world literature [27-33]. In most of these, right coronary artery arises from proximal or mid LAD and courses to the right towards the right AV groove or the acute margin of right ventricle, with the ischaemic symptoms resulting primarily from the stenotic disease in LAD or rarely in RCA [27-31]. Amasyali et al, however, described the intraseptal course of such an anomalously arising RCA from LAD which then turned rightwards sharply to regain the right AV groove and speculated on whether such meandering course was responsible for inferior ischaemia [32]. Hamodraka et al reported the only case of a left anterior descending artery coursing normally along the anterior interventricular groove and continuing as the posterior descending artery along the posterior interventricular groove after going around the left ventricular apex, in the absence of a normally placed right coronary ostium [33]. Kamran et al described, in a patient requiring mitral valve replacement for fulminant endocarditis, the incidental discovery of an anomalous right coronary artery arising from mid LAD, which coursed along the free wall of right ventricle into the right atrioventricular groove, and continued as posterior descending artery. In the only report of its type in world literature, this was associated with a separate small proximal RCA originating from the right coronary cusp, with conus, right atrial and right ventricular branches [34]. John described LIMA graft to LAD in a patient with a stenotic LAD, with an aberrant vessel arising from the LAD, proximal to the stenosis, which passed anterior to the root of pulmonary artery and right ventricle, down the acute margin of the heart, on to the inferior surface to terminate as the posterior descending artery. This was again associated with a normally arising small non-dominant proximal RCA [35].

Shammas et al reported two cases of a single left coronary artery with continuation of circumflex as the distal right coronary artery without stenotic disease [36]. In a series of 8500 consecutive coronary angiographies, Neuhaus et al reported 3 (0.035%) cases of anatomically single left coronary artery with origin of right coronary artery from the AV branch of dominant circumflex artery in the absence of any coronary artery disease or other cardiovascular abnormalities [37]. Tavernarakis described one case of anomalous origin of right coronary artery from peripheral segment of circumflex artery among 3100 selective angiograms performed in the absence of any clinical abnormality [38]. Extramural, straight and large (>1 mm) intercoronary communications with well-defined muscular layer typical of an epicardial coronary artery, as distinct from coronary collaterals, have been described between circumflex and right coronary arteries. These are thought to arise as a result of faulty embryological development which allows existing intercoronary channel to remain patent and maintain large calibre, and have a potential role in protecting myocardium should atherosclerosis develop in either parent vessel [39].

Thus, in a circulation where there is a single left coronary artery and absent right coronary ostium, the right coronary artery tends to originate as a single vessel either from the left main stem or from the LAD or circumflex systems. Yamanaka et al reported 31 cases of single left coronary artery and categorised them into two groups [1]. In Type L-1, the markedly dominant left coronary artery perfuses the entire myocardium and the right coronary artery is a terminal part of left coronary artery. In Type L-2, the ectopic right coronary artery arises either from proximal left coronary artery (A) or from left main trunk (B). Our patient is the first patient, in the entire world literature, with a single left coronary artery and absent right coronary ostium, in whom the right coronary artery has a dual origin. The proximal right coronary artery arose from the LAD, distal to first diagonal but proximal to first septal artery, ran to the right across the RVOT and gained the right AV groove proximal to the acute margin of right ventricle and established an anastomosis with the distal RCA which arose as a continuation of the circumflex artery. This anomalously arising proximal RCA was a small calibre vessel, pursued a meandering course to gain the right AV groove and, interestingly, was free from atherosclerotic disease itself and arose from the LAD proximal to the stenotic lesion in it. The distal RCA arose as a continuation of the circumflex artery in the posterior AV groove after the circumflex artery gave off the obtuse marginal and posterior descending branches, the main trunk of the circumflex artery having a severe stenotic lesion proximal to the origin of the obtuse marginal artery. This distal RCA gave off a branch to the inferior surface of right ventricle and then continued in the right AV groove to a point well proximal to the acute margin of the right ventricle. The anomalous proximal RCA managed to establish a communication with the distal RCA in the AV groove thus completing the classic C loop of the right coronary artery.
